# Analysis of Capped Silver Nanoparticles Combined with Imipenem against Different Susceptibility Profiles of *Klebsiella pneumoniae*

**DOI:** 10.3390/antibiotics12030535

**Published:** 2023-03-08

**Authors:** Inglid Fontoura, Thaís S. Veriato, Leandro J. Raniero, Maiara L. Castilho

**Affiliations:** 1 Bionanotechnology Laboratory, Research and Development Institute, University of Paraiba Valley, São José dos Campos 12244-000, SP, Brazil; 2 Nanosensors Laboratory, Research and Development Institute, University of Paraiba Valley, São José dos Campos 12244-000, SP, Brazil

**Keywords:** silver nanoparticles, Imipenem, *Klebsiella pneumoniae*, carbapenems, susceptibility profiles, resistant bacteria

## Abstract

*Klebsiella pneumoniae* (*K. pneumoniae*) is an opportunistic bacterium that has drawn attention due to its resistance to carbapenem antibiotics. The treatment of patients with severe infections has been challenging. Thus, silver nanoparticles (AgNPs) have been applied for their antimicrobial effects. This work aims to analyze the synergistic effect of the carbapenem antibiotic Imipenem with AgNPs against different susceptibility clinical profiles of *K. pneumoniae*. The silver nanoparticles were synthesized by bottom-up methodology and capped with alpha-lipoic acid. Susceptibility tests were performed using four *K. pneumoniae* strains with different susceptibility profiles to Imipenem. The strains were induced to form a biofilm for 48 h. Crystal violet and Resazurin assays were performed to determine biofilm formation and minimal inhibitory concentration, respectively. The reduction in Imipenem concentration with the association of nanoparticles was found in all strains studied in planktonic form, and the synergism between silver nanoparticles and Imipenem was demonstrated through the analysis of the fractional inhibitory concentration index. The viability percentage was reduced at rates ≥80% in the biofilm analysis, characterized by the minimal biofilm inhibitory concentration. The study’s proposed association resulted in inhibitory effects on different *K. pneumoniae* profiles, both in planktonic forms and biofilm, with peculiar behavior in the Imipenem-resistant profile.

## 1. Introduction

*K. pneumoniae* is an opportunistic Gram-negative bacterium that colonizes the gastrointestinal tract, the nasopharynx of humans, the mucosa of animals, and environments such as water and soil. The species has strains with diverse antimicrobial susceptibility profiles, that are naturally resistant to penicillins and often exhibit acquired resistance. Currently, attention has been drawn to the emergence and dissemination of hypervirulent strains and those resistant to carbapenem antibiotics, with reports of severe infections. Intensivists have been constantly challenged when treating such strains with current antibiotics, increasing the risk of patient complications, length of hospital stay, morbidity and mortality rates, and associated costs [1,2,3,4,5,6]. 

The World Health Organization (WHO) has classified carbapenem-resistant *K. pneumoniae* as a high-priority pathogen for the research, discovery, and development of new antibiotics [7]. The Food and Drug Administration (FDA) also raised the need for new drugs for the treatment of severe bacterial infections and recently approved Recarbrio^®^, the brand name for the combination of the active principles Imipenem, Cilastatin, and Relebactam [8]. Imipenem is a carbapenem antibiotic that inhibits bacterial cell wall synthesis, whereas Cilastatin and Relebactam work to keep Imipenem from being inactivated by renal enzymes and beta-lactamase degradation enzymes, respectively [9]. Despite the efforts, antimicrobial resistance has been considered one of the greatest threats to public health and a subject of constant discussion among the scientific community. The inappropriate use of antibiotics in human medicine and food production has put the population at risk [10,11].

The global pandemic caused by SARS-CoV-2 also had a major impact on antimicrobial resistance due to the excessive administration of antibiotics for the treatment [12]. The consequences are difficult to predict and may vary in the short, medium, and long term with persistent challenges in various spheres of society [13].

Nanotechnology applied to the study of antimicrobials has been relevant due to the use of nanoscale materials, which can result in greater contact and absorption with the bacteria. Among the nanomaterials, silver nanoparticles (AgNPs) have been extensively studied as antimicrobial agents [14,15], which led to their use in medical devices, such as contraceptives, dressings, surgical equipment, and catheters [16]. The combination of AgNPs with other antimicrobial agents has also been reported as enhancing the microbicidal effect [17]. In this context, the present study aims to analyze the synergistic effect of the carbapenem antibiotic Imipenem with AgNPs against different susceptibility profiles of *K. pneumoniae*.

## 2. Results and Discussion

### 2.1. Synthesis and Characterization of AgNPs 

**Figure 1:**
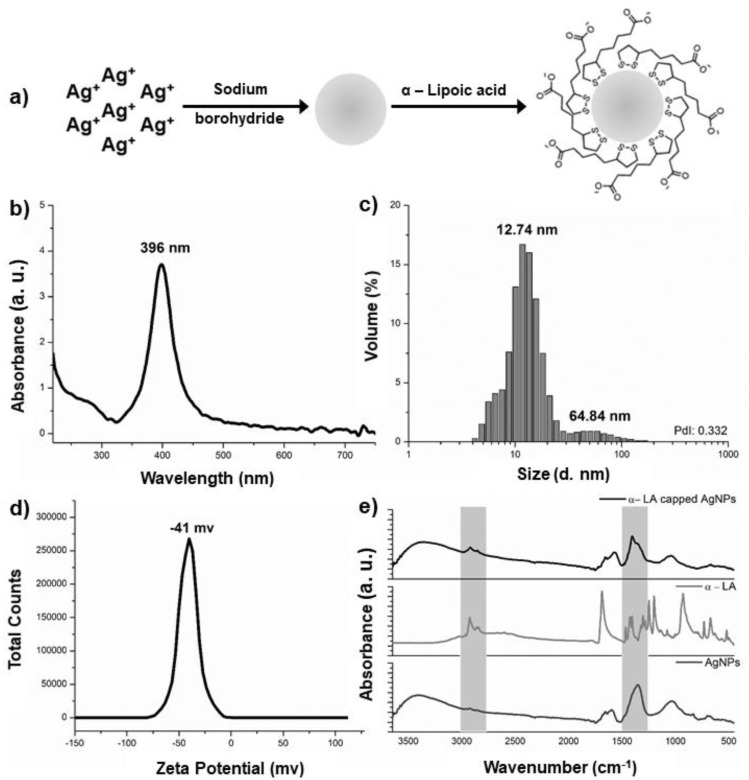
Synthesis and characterization of α-LA capped AgNPs. (**a**) Scheme of synthesis and functionalization of AgNPs; (**b**) UV-Vis absorption spectra of α-LA capped AgNPs; (**c**) Hydrodynamic size measured by DLS of α-LA capped AgNPs; (**d**) Zeta potential of α-LA capped AgNps; (**e**) FT-IR spectra of bare nanoparticles, α-LA, and α-LA capped AgNPs.

The synthesis of AgNPs coated with α-Lipoic Acid (α-LA) used the bottom-up chemical method, in which NaBH_4_ reduced AgNO_3_ to form the colloidal silver solution with yellow color. The exchange of the stabilizing agent occurs by incubating the nanoparticles with the α-LA solution for 24 h, where the bond with the disulfide group present in this molecule suggests a potential capped interaction in the particle (Figure 1a). The α-LA molecule is synthesized by mitochondria [18], leading to reduced cytotoxicity in mammalian cells [19], contributing to the intravenous applicability of the nanopharmaceuticals, as α-LA demonstrates biocompatibility involving endothelium, platelets, and red blood cells [20]. Furthermore, the coating with α-LA not only provides stability to the biological system, in which there is a series of biomacromolecules that bind to the surface of AgNPs, inducing agglomeration and preventing their interaction [21,22], but also promotes an antimicrobial activity, leading to the inhibitory effect that occurs through inducing excessive permeability of the cell membrane with changes in intracellular adenosine triphosphate (ATP) concentration and a decrease in cytoplasmic pH [23]. AgNPs coated with biocompatible materials have recently been studied in vitro and in vivo [24,25]. In vitro, they did not show cytotoxicity, with cell viability values greater than 90% in the cell line L929 [24]. In vivo, the results proved to be an excellent bionanomaterial; histological analyses with sections of various organs of the mice showed no changes in the treated groups compared to the control group, and in the hemocompatibility assay, a low rate of hemolysis was presented [25].

Figure 1b shows the UV–visible (UV-Vis) spectrum of colloids solution capped α-LA, in which a single band centered at 396 nm with a maximum intensity of 3.706 a.u. is observed. The morphology of nanoparticles has a direct correlation with the band absorbance maxima (λMax) in the UV-Vis spectrum and the color of the colloidal solution. Spherical nanoparticles with diameters of up to 20 nm have the λMax between 350 and 400 nm and exhibit yellow shades [26]. Therefore, from the observed results, it is possible to affirm that the morphologies of the AgNPs synthetized are spherical. 

Factors such as the difference in intensity, bandwidth, and shift of the maximum absorption wavelength are instrumental in emphasizing the presence of the coating [21,27]. Furthermore, bare nanoparticles with approximately 10 nm hydrodynamic size exhibit an original surface plasmon band centered around 392 nm [20]. This fact corroborates with results obtained in dynamic light scattering (DLS), where the capped nanoparticles showed a hydrodynamic diameter of 12.74 nm with 93.3% volume distribution and a polydispersity index (PdI) of 0.332 and indicated high antimicrobial efficiency (Figure 1c) [28]. The capped AgNPs showed a slight hydrodynamic increase in the particles, which aids in reducing polydispersion due to the increased stability generated by the coating, given that the zeta potential analysis is −41 mV (Figure 1d), and a value greater than +30 mv or less than −30 mv indicates chemical stability against aggregation of α-LA capped AgNPs [29].

The Fourier-Transform Infrared (FT-IR) spectra highlight the biochemical differences between the bare nanoparticle and capped AgNPs (Figure 1e). The spectra of the capped nanoparticles showed identical frequencies to the synthesized nanoparticles with slight shifts, conformational changes, and band intensities. The bands are found between 3000 and 2800 cm^−1^. The spectrum of the α-LA capped AgNPs nanoparticles show an increase in intensity compared to the same region of the uncapped AgNPs where a discrete shoulder is found. This increase in intensity can be attributed to the contributions of the asymmetric and symmetric stretching (ν) of (C-H) of α-LA at 2926 cm^−1^, 2864 cm^−1^, and 2843 cm^−1^. Contributions from the post-synthesis adsorbed borohydride, such as the H–B–H bending vibrational mode, are observed at 1500–1200 cm^−1^, in addition to the α-LA vibrational modes, such as deformation (δ) (CH_2_) scissoring at 1465 cm^−1^, C–O–H in-plane bending at 1425 cm^−1^, ν(C–H) at 1304 cm^−1^, and ν(C–O)/δ(O-H) out-of-plane at 1248 cm^−1^. The peaks in the hatched region are at 1404 cm^−1^ assigned to ν(C–C), associated with the changes in the 3000–2800 cm^−1^ regions, evidenced by the capping of AgNPs with α-LA [30,31,32,33], corroborating the α-LA molecule spectrum and also the UV-Vis and DLS analyses.

### 2.2. Efficiency of Imipenem Combined with Capped Silver Nanoparticles on K. pneumoniae

The efficacy of an antimicrobial agent depends on the minimal inhibitory concentration (MIC) and minimal bactericidal concentration (MBC) assay results. Table 1 presents the results of the four strains studied with the susceptibility profiles susceptible (Kp-S), intermediate (Kp-I), and resistant (Kp-R) to Imipenem. The MIC of AgNPs in the different profiles of *K. pneumoniae* was 4 × 10^11^ particles/mL, and that of Imipenem performed under the same experimental conditions confirmed the automation profiles (Table 2) according to the breakpoints in the guidelines BrCAST/EUCAST [34]. The combination of AgNPs with Imipenem and the search for a possible synergistic effect led to reduced doses for the treated wells. The inhibition for the ATCC 700603, Kp-S and Kp-I strains was determined by the minimal concentration tested of 0.015 µg/mL, while for the resistant strain (Kp-R), inhibition was achieved with only 0.5 µg/mL combined with 2 × 10^11^ particles/mL of AgNPs. A reduction in Imipenem concentration was observed associating the nanoparticles with all the strains studied, in which the MBC values are very close to the values determined in the MIC; this result was also found by Parvekar and collaborators [35] when using silver nanoparticles against *Staphylococcus aureus*.

The association of AgNPs and Imipenem in the MIC assay reduced antibiotic concentrations by an approximate fold change from 16- to 256-fold (Table 1). The susceptible strains showed a 32-fold reduction in Imipenem concentration, whereas the intermediate strain showed a 256-fold reduction. This fact may relate to the intermediary category that includes isolates with antimicrobial agent MICs that usually approach achievable blood and tissue levels and for which response rates may be lower than for susceptible isolates [36]. Moreover, intermediate strains also include a buffer zone, which should prevent small, uncontrolled, technical factors from causing major discrepancies in interpretations, especially for drugs with narrow pharmacotoxicity margins [36]. The strain with a resistant profile presented a 16-fold reduction when the association between AgNPs and Imipenem was used. Considering that the susceptibility profile of this strain does not favor inhibition with therapeutic doses, this reduction is significant. Several studies propose the combination of antibiotics with AgNPs, including the work of Li et al. [37], who used AgNPs combined with Amoxicillin against Escherichia coli. Deng and colleagues [38] also used several classes of antibiotics associated with AgNPs against multidrug-resistant (MDR) Salmonella typhimurium, and Wan et al. [39] combined AgNPs with Polymyxin B, Rifampicin, and Tigecycline against MDR Acinetobacter baumannii.

Synergistic interactions between AgNPs associated with Imipenem were found in all studied profiles with values of the Fractional Inhibitory Concentration Index (FICI) and the Fractional Bactericidal Concentration Index (FBCI) equal to 0.5 (Table 1), which are commonly used to define the interaction of antimicrobials. Synergy was defined as an FICI or FBCI ≤ 0.5; partial synergy = 0.5 ≤ FICI or FBCI < 1; additive = 1; indifferent = 2 ≤ FICI or FBCI < 4; and antagonism was defined as an index > 4 [40,41]. This result suggests a possible reduction in the viability of bacterial strains at lower antibiotic concentrations. Lopes-Carrizales and co-authors [40] studied the effects of AgNPs combined with Amikacin and Ampicillin on resistant uropathogens in Gram-positive and Gram-negative bacteria. In Gram-negative bacteria, the study showed 2- to 32-fold reductions in Amikacin concentration and 4- to 32-fold reductions in Ampicillin. Regarding the interaction of AgNPs with antimicrobials in different bacteria, synergistic, partially synergistic, and additive combination results have been found [40]. Malawong et al. [41] studied three clinical isolates of *Burkholderia pseudomallei*, a Gram-negative bacteria that was submitted with a combination of silver nanoparticles and different antibiotics, including Ceftazidime, Imipenem, Meropenem, and Gentamicin. The associations between the drugs showed fold changes of 2 to 16 times and synergistic combinations [41].

**Figure 2:**
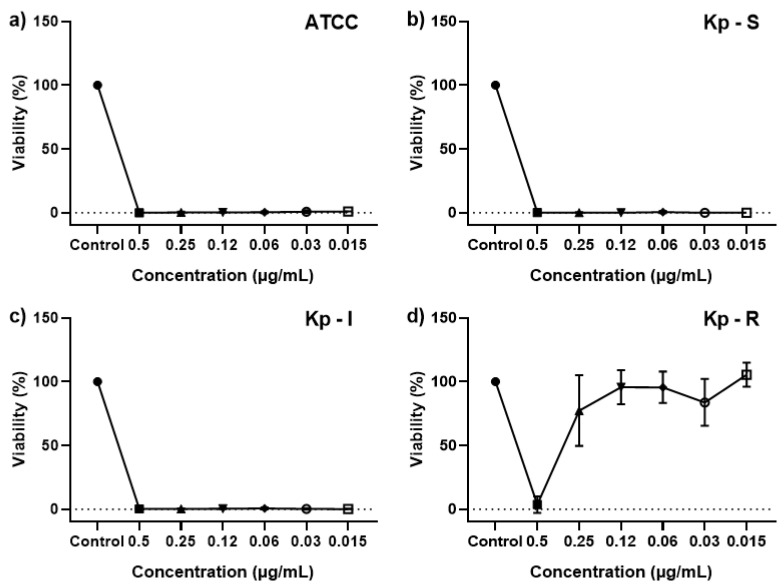
Metabolic activity of the Resazurin assay in planktonic cells of *K. pneumoniae*. (**a**) ATCC; (**b**) Kp-S; (**c**) Kp-I; and (**d**) Kp-R. Data are expressed as the mean ± Standard Deviation (SD) of triplicate determinations.

An MIC is usually associated with laboratory investigations of antibiotic susceptibility profiles against microorganisms. However, nanoparticle characteristics, particularly opacity and insolubility in culture medium, can hinder MIC assay and application in clinical laboratories that rely on the visual observation of turbidity [42]. In this context, because it is a simple and high-throughput method, the Resazurin assay was used to quantify the performance of the nanomaterial associated with the antibiotic, allowing the determination of the MIC to be confirmed. Figure 2 shows the viability of planktonic cells subjected to action with AgNPs combined with Imipenem. The metabolically active bacteria convert the weakly fluorescent redox dye Resazurin into the fluorescent product Resorufin [43]. Therefore, the relative fluorescence units (RFU) were used to calculate the viability percentage. The strains with susceptible and intermediate profiles do not show growth in any concentration of the association tested. In contrast, the strain with a resistant profile showed growth inhibition only at a concentration of 0.5 µg/mL combined with nanoparticles, proving the reduction in bacterial viability and correlating with the MIC results.

**Figure 3:**
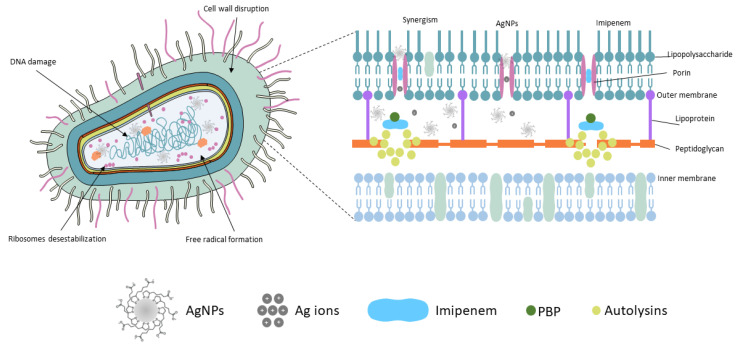
Schematic representation describing the interaction and antimicrobial action of silver nanoparticles, Imipenem, and the agents’ association on the Gram-negative bacterium.

AgNPs and Imipenem are bactericidal agents and present different mechanisms of action. The results found in the MIC, MBC, and Resazurin assays demonstrate the synergistic effect between the nanoparticles and the antibiotic proven by FICI and FBCI, considering that each agent’s isolated concentration was not enough to conduct the bacteriostatic or bactericidal effect. Thus, the action mechanism and the synergistic effect of AgNPs and Imipenem on bacteria are presented in Figure 3.

The effect of AgNPs on bacteria can be explained initially by adhesion to the bacterial cell wall surface, penetration into the cell, and disruption of organelles and intracellular biomolecules. Protein synthesis is altered, destabilizing the cellular outer membrane composition with the induction of reactive oxygen species’ (ROS) release, causing cellular toxicity, oxidative stress, free radical formation, and damage to mitochondria and DNA. This antimicrobial effect is also potentiated with Ag^+^ ions. Ions and nanoparticles smaller than 2 nm penetrate cells through porin channels in the outer membrane of Gram-negative bacteria by binding to proteins and nucleic acids, causing a confluence of bacterial structural changes [17,44,45]. In contrast, Imipenem has a β-lactam ring in its structure that gives them the ability to bind to and inactivate relevant transpeptidases, known as penicillin-binding proteins (PBPs), which are responsible for the elongation and cross-linking of the bacterial cell walls’ peptidoglycan. In response to the inactivation of the PBPs, the autolysins are activated, interfering with cell wall formation. Carbapenems, such as Imipenem, can bind to a specific PBP (PBP-1), resulting in faster lysis than other beta-lactams and higher bactericidal activity [46,47,48]. The association of these events leads to cell death regardless of the susceptibility profile of the microorganism.

### 2.3. Antimicrobial Effects of Imipenem Combined with AgNPs on Biofilm K. pneumoniae

**Figure 4:**
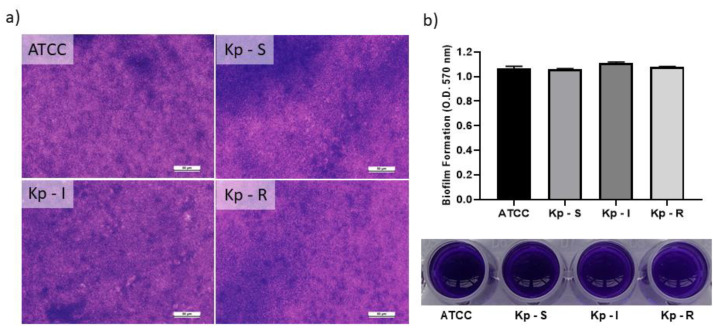
*K. pneumoniae* biofilm formation: (**a**) The crystal violet assay was used to visualize biofilm formation on polystyrene plates. The scale bars represent 50 μm, and the magnification is ×200; (**b**) graph of the optical density reading (OD_570_) expressed as mean ± SD of the biofilms with the association of the photograph of the respective wells before the OD measurement.

The crystal violet assay was carried out to determine the biomass during the biofilm production by *K. pneumoniae* (Figure 4). The biofilm formation ability of the four strains, in which the ATCC strain formed a biofilm with moderate adhesion and optical density at 570 nm (OD_570_) values of 1.07 ± 0.02 after 48 h of incubation, was analyzed (Figure 4b). When analyzing the clinical strains with a different susceptibility profile, it was observed that the biofilm formation showed moderate adhesion regardless of the profile, with OD_570_ values for Kp-S of 1.08 ± 0.01, Kp-I of 1.11 ± 0.01, and for Kp-R of 1.06 ± 0.01 (Figure 4b). Micrographs provided evidence that corroborated biofilm formation activity (Figure 4a).

The optical density of the biofilm can reflect its ability to adhere compared to the optical density of the control group [49]. Moderate adhesion can be interpreted as positive biofilm production according to the analysis proposed by Stepanovic et al. [50]. The quantification of biofilm biomass is presented in the study of Oleksy-Wawrzyniak and co-authors [51], where they analyzed the adherence of 118 isolates of *K. pneumoniae* on polystyrene plates from the crystal violet assay, and 51% of isolates showed biofilms with moderate adherence. This study also demonstrated *K. pneumoniae* ATCC 700603 adherence with the obtained value of OD 1.00 unit ± 0.35, corroborating the results shown in Figure 4b.

**Figure 5:**
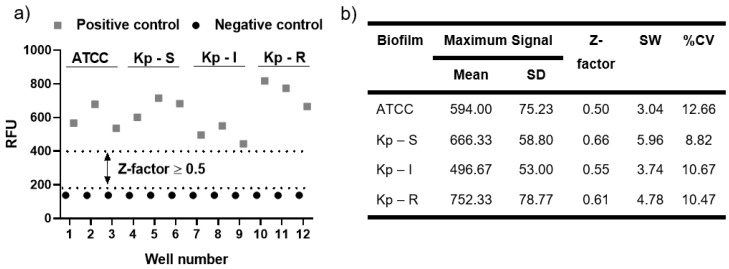
Indicator of assay quality of biofilm formation of *K. pneumoniae*. (**a**) Distribution chart of the positive and negative controls of the studied biofilms and the Z-factor presentation; (**b**) Mean and SD in RFU of the positive control biofilms, with the determination of the Z-factor, signal window (SW), and percent coefficient of variation (%CV). The negative control had a mean of 137.67 RFU with an SD of 0.58 RFU.

The results of biofilm formation by the Resazurin assay met the criteria established by the Z-factor, which is an indicator of assay quality (Figure 5) [52,53,54]. The *K. pneumoniae* biofilms indicate a high-quality assay exhibiting a wide separation between positive and negative control and acceptable data variability (Figure 5a). The values obtained demonstrate data suitable for bioassay (Figure 5b), where the Z-factor is greater than 0.5, the signal window is greater than 2, and the coefficient of variation is less than 20%. Thus, a high degree of precision and sensitivity in the assay is essential, considering the variability of measurements [54].

**Figure 6:**
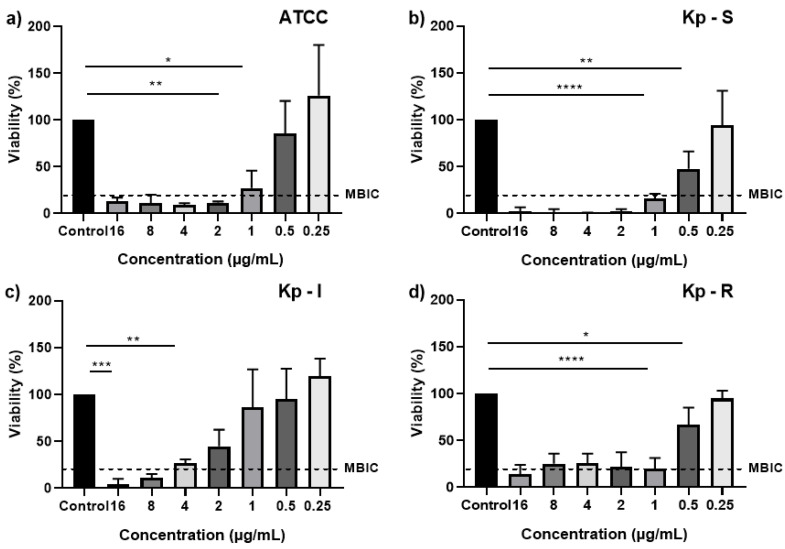
Viable cells in biofilms determined via the Resazurin assay after administration with AgNPs combined with Imipenem. (**a**) ATCC; (**b**) Kp-S; (**c**) Kp-I; and (**d**) Kp-R. Data are expressed as mean ± SD. * *p* < 0.05, ** *p* < 0.01, *** *p* < 0.001, **** *p* < 0.0001.

The impacts on the inhibition of biofilms formed with moderate adhesion were demonstrated by combining AgNPs (2 × 10^11^ particles/mL) and Imipenem (16 µg/mL to 0.25 µg/mL) followed by the Resazurin assay. The percentage of biofilm viability was determined, followed by normality and lognormality tests for the appropriate statistical test analysis. A regular distribution in the quantile–quantile (QQ) plot graphs was observed, and the Shapiro–Wilk test determined approval of the normality test (alpha = 0.05). The results of the one-way ANOVA are presented in Figure 6, in which significant differences are found between the tested concentrations in the different susceptibility profiles. At the concentration of 0.25 µg/mL of the antibiotic-associated AgNPs, there was an increase in the viability of bacteria present in the biofilm compared to the control group, especially in the susceptible (Kp-S) and intermediate (Kp-I) profiles. Sub-inhibitory concentrations of antibiotics have been reported as increasing their metabolic activity. There is growing evidence that bacteria respond specifically and defensively to these concentrations. In particular, Gram-negative bacteria can respond with numerous strategies to combat antibiotics and, more recently, AgNPs [22,55,56,57].

The viability reduction was found with an increased Imipenem concentration associated with AgNPs in all biofilms. The clinical strain with a susceptible susceptibility profile (Kp-S) showed a viability < 2% for 2 µg/mL concentrations up to 16 µg/mL. The strain with a resistant profile (Kp-R) showed viability of 25.4% when the MIC alone of the antibiotic was used at 8 µg/mL.

The minimal biofilm inhibitory concentration (MBIC) is defined as the concentration that inhibits ≥80% of the metabolic activity of each treated biofilm compared to the positive control [58]. Thus, it can be defined that the MBIC for the ATCC strain was 2 µg/mL, and Kp-S was set at 1 µg/mL for the clinical strain. However, for clinical strains Kp-I, increasing concentrations of Imipenem associated with AgNPs showed a dose-dependent aspect of growth inhibition, with the MBIC defined as 8 µg/mL to contain at least 80% of viability. However, when analyzing the biofilm with a resistant profile (Kp-R) regarding the inhibitory effect generated by the association of AgNPs and Imipenem, a behavior similar to that of the susceptible biofilm with an MBIC equal to 1 µg/mL was observed presenting an eight-fold reduction compared to the MIC value of the antibiotic alone, whereas for the susceptible profile, there was an increase in concentration for biofilm inhibition compared to planktonic strains. This find could be attributed to a possible synergistic effect generated by both antibacterial agents, implying that the action mechanism causes the resistant biofilm to lose its inhibition effectiveness to Imipenem. Moreover, AgNPs can easily penetrate the biofilm, and their association with the antibiotic increases the anti-biofilm activity [17,59].

Overall, this study demonstrated that Imipenem has a synergistic effect with AgNPs in planktonic cells. The association reduced the concentration of Imipenem in MIC tests, which is an assay that guides the appropriate antibiotic choice by the intensivist and infectious disease specialist for administration to patients. A lower dose of Imipenem associated with AgNPs may reduce the drug’s neurotoxic and nephrotoxic side effects. Furthermore, it was observed that the association of the antibiotic with AgNPs exhibited an inhibitory effect on biofilms with similar concentrations and even reduced the MIC.

## 3. Material and Methods

### 3.1. Synthesis of Silver Nanoparticles

AgNPs were synthesized using the bottom-up chemical method by reducing silver nitrate (AgNO_3_; Sigma-Aldrich (St. Louis, MO, USA), 209139) by sodium borohydride (NaBH_4_; Sigma-Aldrich, 452882) to obtain the colloidal solution. Thus, all glassware and magnetic stirring bars used were cleaned with aqua regia with a 1:3 ratio of nitric acid (HNO_3_, ≥ 65%; Sigma-Aldrich, 84378) and hydrochloric acid (HCl, 37%; Neon (São Paulo, Brazil), 02618) to remove contaminating metals and then rinsed in ultra-pure water prior to use. In an ultra-thermostatic bath (Marconi, MA-184, Piracicaba, Brazil), a 30 mL aliquot of the 1 mM NaBH_4_ solution was cooled and stirred for 1 h at 0 °C. Subsequently, 10 mL of 2 mM AgNO_3_ was added to the solution, and after seven minutes in the dark, colloidal silver with a yellow coloration was observed [60]. The solution was left in the light for 24 h. Subsequently, α-LA (Sigma-Aldrich, T5625) at a concentration of 7.75 mM was introduced into the colloidal solution under constant stirring (IKA-MS3, Staufen, Germany) for 24 h to stabilize the AgNPs in a biological medium. The chemical by-products of the synthesis were removed by centrifugation at 15,000 rpm for ten minutes (Multifuge X1R; Thermo Scientific, Langenselbold, Hessen, Germany), the supernatant was discarded, and the pellet was resuspended in ultrapure water. The sample was stored in the dark and characterized by UV-Vis, DLS, and FT-IR spectroscopy.

#### 3.1.1. UV-Visible Spectroscopy

The UV-Vis absorption spectra were acquired on a DeNovix DS-11 spectrophotometer (DeNovix Inc., Wilmington, DE, USA) with a resolution of 1.5 nm. The microvolume mode’s spectral region of 220–750 nm was used with 2 µL of each sample in the pedestal. The data were plotted using OriginPro, version 8.5.1 (Origin Lab., Northampton, MA, USA).

#### 3.1.2. Dynamic Light Scattering (DLS)

The hydrodynamic diameter of the nanoparticles and the Zeta potential were determined by the Zetasizer Nano ZS90 (Malvern Instruments, Worcestershire, UK). The average diameter and PdI analyses were performed in the size mode, using a 400 µL aliquot of AgNPs deposited in a disposable polystyrene cuvette (ZEN0118). The three measurements of each sample at 90° were taken at 25 °C with an equilibration time of 120 s. Zeta potential was measured in zeta mode using the same parameters cited above with 1 mL of AgNPs deposited in disposable, folded capillary cells (DTS 1070). The values correspond to the average of three independent measurements.

#### 3.1.3. Fourier Transform Infrared (FTIR) Spectroscopy

The mid-infrared spectra were collected on the Spectrum 400 FT-IR/FIR equipment (Perkin Elmer, Waltham, MA, USA) using a GladiATR (PIKE Technologies, Fitchburg, WI, USA) accessory. The average spectrum was obtained in the range of 4000–450 cm^−1^ by collecting three spectra with 16 scans in absorbance mode at a resolution of 4 cm^−1^. Spectral normalization was performed in OPUS version 4.2 (Bruker, Ettlingen, Germany) and plotted in OriginPro version 8.5.1 (Origin Lab., USA).

### 3.2. Dilution of the Antibiotic Imipenem

The antibiotic Imipenem (C_12_H_17_N_3_O_4_S-H_2_O, Sigma-Aldrich, PHR-1796) was previously diluted in phosphate buffer.

### 3.3. Bacterial Strain and Culture Conditions

In this study, the strain standard (ATCC 700603) and clinical strains (Kp-S, Kp-I, and Kp-R) of *K. pneumoniae* with different susceptibility profiles were evaluated (Table 2). Clinical isolates were obtained from Laboratório Oswaldo Cruz (Kp-S and Kp-I) and the Hospital de Clínicas Sul (Kp-R), located in São José dos Campos, SP, Brazil. The identification and susceptibility profiles of the strains were performed using the BD Phoenix equipment (Becton Dickinson, Franklin Lakes, NJ, USA). Bacteria on the storage surface used brain heart infusion (BHI) broth (Oxoid, Basingstoke, Hampshire, England, CM 1135) with 20% glycerol (LGC, 13-1325-10) in the deep ultra-freezer (−80 °C). Strains were reactivated in BHI broth, being incubated in a bacteriological oven (Heratherm; Thermo Scientific, Dreieich, Hesse, Germany) for 24 h at 35 °C (±2 °C) and seeded onto BHI agar (Oxoid, CM 1136) for 24 h at 35°C (±2 °C). The colony morphology and Gram staining were analyzed.

### 3.4. Minimal Inhibitory Concentration and Minimal Bactericidal Concentration Assay

The broth microdilution method was used to determine the MIC according to International Organization for Standardization (ISO) standard 20776-1 [61]. The Mueller-Hinton broth (MHB—Himedia, Dindhori, India, M391) was dispensed in a 96-well plate, and different concentrations of the antibiotic Imipenem were tested, following a serial dilution starting from a concentration of 16 µg/mL to 0.007 µg/mL. The concentration of AgNPs in the association was set at 2 × 10^11^ particles/mL, with a concentration below the MIC of AgNPs alone. The inoculum was prepared in saline solution (0.9%) initially on the McFarland 0.5 scale (1 × 10^8^ CFU/mL) (Densichek plus; BioMerieux, Craponne, France), followed by a 1:20 dilution (5 × 10^6^ CFU/mL) with a concentration of 5 × 10^5^ CFU/mL at the end of the test. Sterility control (Imipenem, AgNPs, and MHB) and growth control were also included in the microplate. The plates were incubated at 35 °C (± 2 °C) for 18 h. After incubation, there was a visual investigation of the MIC, which was confirmed by reading the optical density by absorbance at a wavelength of 600 nm (OD_600_) in the Synergy HTX spectrophotometer (BioTek Instruments, Winooski, VT, USA). The MIC was defined as the minimal concentration capable of inhibiting bacterial growth. The experiment was carried out in triplicate.

The MBC was determined by the microdrop method, in which a 10 μL volume of the MIC concentrations assay was deposited on the surface of BHI agar. After absorbing the microdrop, the plates were inverted and incubated at 35 °C (±2 °C) for 24 h. The absence of bacterial growth on the agar indicated the bactericidal effect.

### 3.5. Quantification of Planktonic Cell Viability

In the Resazurin assay (Resazurin sodium salt—Sigma-Aldrich, R7017), 2 µL of the reagent at 6.75 mg/mL was added to each well; then, tested in the MIC and incubated for two hours in a bacteriological oven. The whole procedure was carried out in the dark. The relative fluorescence units (RFU) reading was performed with a Synergy HTX spectrophotometer (BioTek Instruments, USA), with excitation at 528 nm and emission at 645 nm. At least three independent replications were performed for each sample. The percentage of the viability of grown planktonic cells relative to the positive control (PC) was determined according to Equation (1), in which the absorption value of the material control (MC) group was subtracted:

(1) $$Viability\left(\%\right)=\frac{RFU_{Sample}-RFU_{MC}}{RFU_{PC}-RFU_{MC}}\times 100\%$$

### 3.6. Biofilm Formation

Biofilms were formed using 100 µL of BHI broth and 100 µL of the standardized inoculum in saline (0.9%) at 10^7^ CFU/mL in polystyrene 96-well microplates. These microplates were incubated at 35 °C (±2 °C) in a bacteriological oven. The medium was renewed every 24 h during incubation. The biofilm formation occurred within 48 h.

#### 3.6.1. Determination of Biofilm Formation by the Crystal Violet Assay

The crystal violet assay was performed to classify the biofilms of *K. pneumoniae*. After the content of each well was removed and gently washed twice with a sterile saline solution of 0.9% to remove planktonic and loosely-bound cells, 200 µL of methanol was added for 20 min to fix the biofilm. Methanol was then removed, and the plate was incubated to dry at 35 °C (±2 °C) for 24 h. It was stained with 1% (wt./vol) crystal violet solution for 5 min at room temperature, and subsequently, the excess stain was washed off twice with sterile saline solution (0.9%). The biofilms were resuspended in 200 µL of acetic acid 33% and measured by absorbance at a wavelength of 570 nm (OD_570_) in SpectraCount (BS10001; Packard BioScience Company, Downers Grove, IL, USA) to determine the biofilm biomass.

The OD defines the adhesion capacity of the biofilm. However, the cut-off value (ODc) should be established from the arithmetic mean of the absorbance of the negative controls with the addition of three times the SD. Thus, we classified the formed biofilms as no biofilm production (OD ≤ ODc), weak biofilm production (ODc < OD ≤ 2ODc), moderate biofilm production (2ODc < OD ≤ 4ODc), and strong biofilm production (4ODc < OD) [62].

#### 3.6.2. Determination of Biofilm Viability by the Resazurin Assay

In the biofilm formation, the culture medium was then removed and 200 µL of AgNPs (2 × 10^11^ particles/mL) combined with different Imipenem concentrations (16 µg/mL to 0.25 µg/mL) were added to the wells for 24 h at 35 °C (± 2 °C). The Resazurin colorimetric assay was used to determine the viability of the biofilm of *K. pneumoniae*. Biofilms were washed with sterile phosphate-buffered saline (PBS) to remove planktonic and loosely bound cells after the treatment with AgNPs combined with Imipenem. Then, 200 µL of sterile PBS was used for resuspension along with the addition of 4µL of Resazurin (6.75 mg/mL) for 6 h at 35 °C (±2 °C) in a bacteriological oven. The RFU reading was performed on the Synergy HTX spectrophotometer (BioTek Instruments, USA) with excitation at 528 nm and emission at 645 nm. At least three independent replications were performed for each sample. Statistical analysis was performed by applying the Z-factor, signal window (SW), and the percent coefficient of variation (%CV) equations in RFU using Excel software (Microsoft Office Professional Plus 2016). The percentage of the viability of the grown biofilm relative to the positive control (PC) was determined according to Equation (1), in which the absorption value of the material control (MC) group was subtracted. The normality and lognormality tests were performed; data distribution analysis (QQ plot) with an evaluation of the Shapiro–Wilk test, followed by the application of the analysis of variance, one-way ANOVA, complemented by Tukey’s test, considering a significance of *p* ≤ 0.05 in GraphPad Prism 8.0.1 (GraphPad Software San Diego, CA, USA).

## 4. Conclusions

The association of the carbapenem antibiotic Imipenem with the capped AgNPs α-LA showed a synergistic and inhibitory effect against different susceptibility profiles of *K. pneumoniae* in planktonic cells and biofilms. Imipenem action was not inhibited when associated with nanoparticles in a resistant clinical strain (Kp-R), a characteristic observed by the bacterial response with an increasing antibiotic concentration to the detriment of AgNPs fixed concentration. As a result, a synergistic combination of antimicrobial drugs and AgNPs has offered an excellent solution to this health problem, addressing the need for new technologies to act against MDR microorganisms.

## Figures and Tables

**Table 1:** Minimal inhibitory concentration and minimal bactericidal concentration assay data of silver nanoparticles, Imipenem, and their association against different profiles of *K. pneumoniae*.

Strains	MIC			Fold Change	FICI	MBC			Fold Change	FBCI
	AgNPs	IMP	AgNPs + IMP			AgNPs	IMP	AgNPs + IMP		
ATCC	4	0.5	0.015	32	0.5	4	0.5	0.015	32	0.5
Kp-S	4	0.5	0.015	32	0.5	4	0.5	0.015	32	0.5
Kp-I	4	4.0	0.015	256	0.5	4	4.0	0.015	256	0.5
Kp-R	4	8.0	0.500	16	0.5	4	16.0	0.500	32	0.5

AgNPs (silver nanoparticles): 10^11^ particles/mL; Imipenem (IMP): µg/mL and AgNPs + IMP: 2 × 10^11^ particles/mL + Imipenem concentration in µg/mL.

**Table 2:** Antibiotic susceptibility profiles of *K. pneumoniae* clinical isolate strains isolated from catheter tip (Kp-S and Kp-I) and urine sample (Kp-R).

Antimicrobial	Kp-S	Kp-I	Kp-R
	SIR	SIR	SIR
Ampicillin	R	R	R
Ampicillin/Sulbactam	R	R	R
Piperacillin/Tazobactam	R	R	R
Cefuroxime	R	R	R
Cefoxitin	R	R	R
Ceftazidime	R	R	R
Ceftriaxone	R	R	R
Cefepime	R	R	R
Ertapenem	R	R	R
**Imipenem**	**S**	**I**	**R**
Meropenem	R	R	R
Amikacin	S	S	S
Gentamycin	S	S	S
Ciprofloxacin	R	R	R

S = Susceptible; I = Intermediate; and R = Resistant.

## Data Availability

Data is contained within the article.
